# Elevated Long Term Resting Heart Rate Variation is Associated with Increased Risk of All-cause Mortality in Northern China

**DOI:** 10.1038/s41598-017-07309-2

**Published:** 2017-08-14

**Authors:** Binhao Wang, Huihua Li, Xu Han, Yiheng Yang, Yue Chen, Wenyu Li, Xiaolei Yang, Aijun Xing, Yanxiu Wang, Tesfaldet Habtemariam Hidru, Shouling Wu, Yunlong Xia

**Affiliations:** 1 Department of Cardiology, First Affiliated Hospital of Dalian Medical University, Dalian, Liaoning China; 2 Department of Cardiology, Kailuan General Hospital, Tangshan, Hebei China; 3 Arrhythmia Center, Ningbo First Hospital, Ningbo, Zhejiang China; 4 School of Public Health, Dalian Medical University, Dalian, Liaoning China

## Abstract

Elevated resting heart rate (RHR) predicts all-cause death. However, the relationship between RHR variation over years and mortality are still unknown. We aimed to analyze the association between RHR variation and all-cause mortality in the general population without cardiovascular diseases. A total of 46,873 subjects were included from the Kailuan Study (2006–2011). RHR readings were taken during three separate examinations and the RHR variation was defined using the standard deviation (RHR-SD) and the coefficient of variation. Participants were divided into four groups according to the quartiles of RHR-SD. All subjects were followed for a median of 49.4 months from the date of the 3^rd^ examination to December 31, 2014. Up until the follow-up examinations, 973 (2.08%) participants had died. In a multivariate analysis, adjusting for variables potentially associated with death, the highest quartile of RHR-SD remained an independent predictor of all-cause mortality (Hazards ratio = 1.43, 95% confidence interval 1.18–1.74, *P* < 0.001). These findings suggest that an elevated long-term RHR variation is an independent risk marker for all-cause mortality in the general population without known cardiovascular diseases.

## Introduction

Heart rate is one of our vital signs and varies according to the body’s physical needs. Thus far, various studies have demonstrated a significant relationship between resting heart rate (RHR) and all-cause mortality^1, 2^. Large epidemiologic investigations further substantiate these observations in men and women with and without diagnosed cardiovascular diseases^3–5^. Pathophysiological investigations suggest that a relatively high RHR has direct detrimental effects on the progression of various cardiovascular diseases, including coronary atherosclerosis, myocardial infarction, ventricular function and arrhythmias^6^. A previous study presented that risk of death increased with a heart rate above 60 beats per minute^7^; however, there is a lack of evidence on the dose–response between resting heart rate and all-cause mortality.

Recently, Floyd *et al*. reported that the variation in RHR over a period of several years represents a potential predictor of long-term mortality among older persons free of cardiovascular disease^8^. However, there is no independent study that investigated the relationship between RHR variation and all-cause mortality with a broad statistical analysis that entertains extensive adjustment for potential confounding factors in the general population. Therefore, we conducted a prospective cohort study involving the general population without major cardiovascular diseases (CVDs) to assess the risk of all-cause mortality associated with long term RHR variation.

## Methods

### Study design and population

**Figure 1:**
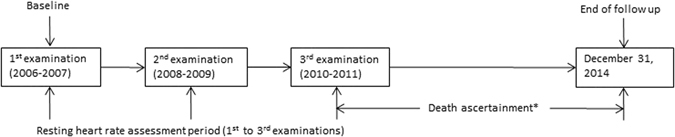
Study design evaluating the association between long term resting heart rate variation and all-cause mortality in the Kailuan Study. Participants were followed up for a median of 49.4 months.

The Kailuan study is a large contemporary population-based, prospective cohort study carried out in Kailuan community in Tangshan City in the Hebei Province of China. The design and methods of the study have been described in detail in the previously published report^9^. A total of 101,510 participants (81,110 men and 20,400 women; aged 18–98 years) are managed in the Kailuan study profile. Figure 1 illustrates the RHR assessment and follow-up period. The 1^st^ examination was carried out from June 2006 to October 2007. Follow-up examinations were performed every two years (2^nd^ examination, 2008–2009; 3^rd^ examination, 2010–2011). The study participants were followed for the endpoint event (death) between 3^rd^ examination and December 31, 2014. To prove that RHR variability assessed over the long period of time was not a consequence of deteriorating health and appearance of diseases that were directly affecting prognosis, clinical characteristics were assessed at the end of repeated ECG examinations (3^rd^ examination, 2010–2011). The subjects who attended 3 consecutive examinations with complete electrocardiography (ECG) recordings were eligible for this study. All subjects with physician-diagnosed CVDs (including history of myocardial infarction/stroke, congestive heart failure and atrial fibrillation/flutter), had an implanted pacemaker and subjects under the use of β blockers and non-dihydropyridines medication through the entire period of RHR assessment (1^st^, 2^nd^, and 3^rd^ examinations) were excluded from this study. The study was conducted in compliance with the law protecting personal data in accordance with the guidelines of Helsinki declaration. The study was approved by the Ethics Committee of Kailuan General Hospital and informed consent was obtained from all participants.

### Assessment of long-term variation in resting heart rate

After participants had been comfortably supine for 5 min in a quiet room, RHR was obtained using a 10-second 12-lead resting supine ECG from every participant between 6:00 AM and 9:00 AM (before breakfast). The standard deviation (SD), coefficient of variability (CV), and average real variability (ARV) of the RHR over the first 3 examinations were calculated by using the following formulae: ${\rm{S}}{\rm{D}}=\sqrt{\frac{\sum (RH{R}_{i}-\bar{RHR}{)}^{2}}{n-1}}$, ${\rm{CV}}=(\frac{SD}{\overline{RHR}})\times 100 \%$, and ${\rm{ARV}}=\frac{1}{n-1}\sum _{i=1}^{n-1}|RH{R}_{i+1}-RH{R}_{i}|$. *RHR*_*i*_ is the RHR of the participant at the examination. $\overline{RHR}$ is the average RHR of the participant across examinations. To properly account for the changed clinical status and assess whether the impact of changes in RHR was independent from clinical characteristics, prediction of outcome was assessed based on the clinical characteristics demonstrated at the last ECG assessment.

### Collection and definitions of potential covariates

A standardized interview was conducted during each examination to collect data on health-related lifestyle, disease history, family history and use of anti-hypertensive drugs including angiotensin-converting-enzyme inhibitor (ACEI), angiotensin receptor blocker (ARB), calcium channel blockers (CCB) only dihydropyridines, diuretics, and others (such as α blockers and traditional Chinese medicine, etc.). Blood pressure (BP) was measured in a sitting upright position after waiting 5 min. Body mass index (BMI) was calculated as the weight (kg) divided by height squared (m^2^). Fasting (>8 h) blood specimens were collected and were biochemically examined for the concentration of fasting plasma glucose (FPG) and C-reactive protein (CRP). Elevated CRP was defined as the level ≥2 mg/L. Standard enzymatic methods were used to measure serum total cholesterol (TC) and high-density lipoprotein (HDL). Smoking status was grouped into three categories: current smoker (history of cigarette smoking during the past year), former smoker (history of ever smoking) and never smoker (participants who had never smoke in their lifetime). Physical exercise was divided into two categories: sedentary/moderate activity for <4 h per week and high/intense activity for ≥4 h per week. Diabetes mellitus (DM) was defined as FPG ≥7.0 mmol/L or random plasma glucose ≥11.1 mmol/L or a self-reported diabetes history with the current use of anti-diabetes medication. Hypertension was defined as systolic BP (SBP) ≥140 mmHg and/or diastolic BP (DBP) ≥90 mmHg, or a self-reported history of hypertension and currently undergoing a treatment with antihypertensive medication.

### Mortality follow-up

All participants were followed for the endpoint event (death) from the date of 3^rd^ examination to December 31, 2014. A total of 973 deaths were reported from the regional health office and local hospital records. Death was ascertained by professional doctors through surveying discharge summaries, medical records from hospitals or medical insurance companies. Also, death certificates were regularly screened from state vital statistics offices annually for the sites included under the Kailuan study.

### Statistical analysis

Test variables were summarized according the following system: quartiles (Q) of standard deviation (SD) of RHR (RHR-SD), mean ± SD for continuous variables and percentiles for categorical variables. The study population were stratified into quartiles based on the RHR-SD (Q1 group: participants with the RHR-SD <3.6 bpm, Q2: participants with the RHR-SD range of 3.7–5.8 bpm, Q3 group: participants with the RHR-SD range of 5.9–8.5 bpm, and Q4 group: participants with RHR-SD >8.5 bpm). Quartiles were tested for differences using ANOVA and χ^2^ test for continuous and categorical data respectively. The association between RHR-SD with participant characteristics was performed by linear regression analysis. Demographic and clinical characteristics included in the analysis were age, gender, BMI, current smoker, sedentary/moderate activity, SBP, DBP, mean RHR, TC, HDL, FPG, elevated CRP, DM, and antihypertensive medication classes. The initial model was adjusted for age and gender, and the subsequent model was adjusted for all variables associated with RHR-SD in the age- and gender-adjusted model.

Survivor functions were estimated for each quartile using the Kaplan-Meier method and statistically evaluated using a Log-rank test of trend. We used Cox proportional hazards model with the lowest quartile as the reference, to calculate unadjusted and adjusted hazard ratios (HRs) and corresponding 95% confidence intervals (CI) for the occurrence of death. Moreover, the association of RHR-SD, modeled as a continuous variable, with all-cause mortality was evaluated using Cox proportional hazard models. The initial model was unadjusted; and the subsequent model was adjusted for age and gender. The multivariable adjusted model included age, gender, smoking, BP, mean RHR, CRP, and anti-hypertensive drugs. The analyses conducted based on the RHR-SD were repeated after remodeling quartiles by CV of RHR (RHR-CV) and ARV of RHR (RHR-ARV) in order to examine the consistency of the results. All statistical analyses were conducted using SAS 9.3 (SAS Institute; Cray, NC). A *P* value of less than 0.05 was considered statistically significant.

### Data Availability

The datasets generated and analyzed during the current study are available from the corresponding author upon reasonable request.

## Results

### Clinical characteristics

A total of 101,510 participants were originally included in this study. We excluded 52,513 participants without baseline data and 1,065 participants with a prior history of coronary artery disease, myocardial infarction or stroke. Additionally, 619 participants were diagnosed with stroke/myocardial infarction during follow-up;232 participants with history of β blocker treatment;192 participants whose ECG readings with atrial fibrillation or flutter at examination 1, 2 or 3; and 16 participants received pacemaker before examination 3 were excluded from this study. Therefore, 46,873 participants (36,563 men, and 10,310 women) were included in this analysis.

The clinical characteristics of the participants by RHR-SD quartile at the 3^rd^ examination are shown in Table 1. The 25%, 50%, and 75% percentile of RHR-SD were 3.6 bpm, 5.8 bpm, and 8.5 bpm, respectively. The proportion of males was larger than females in all groups. The values of the mean SBP, DBP and RHR were lower in the lowest quartile (Q1) than in Q2-Q4. The proportion of elevated CRP and the mean FPG were higher in the highest quartile (Q4) than in Q1-Q3. There was a higher proportion of ACE inhibitor medication in Q1, 3 and 4 than Q2, indicating that subjects in the higher or lower quartiles of RHR-SD received more treatments of ACE inhibitor. There were no significant differences in age, BMI, current smoker category, high or intensive activity, total cholesterol, HDL, diabetes mellitus, and the use of ARB, CCB and diuretic between the different quartiles.

**Table 1:** Clinical characteristics of participants divided by quartiles of standard deviation in resting heart rate * (n = 46,873).

	Quartile of standard deviation in resting heart rate, bpm				*P* for trend
	Quartile 1 (<3.6)	Quartile 2(3.6–5.8)	Quartile 3(5.8–8.5)	Quartile 4 (≥8.5)	
Participants (n)	11,676	12,230	10,912	12,055	—
Age, mean (SD), years	52 (11)	52 (11)	53 (12)	53 (12)	<0.001
Men (%)	76	77	79	81	<0.001
Body mass index, mean (SD), kg/m^2^	25 (3)	25 (3)	25 (3)	25 (3)	0.324
Current smoker (%)	34	34	36	35	0.044
High physical activity (%)	13	13	13	13	0.625
Systolic blood pressure, mean (SD), mmHg	128 (16)	129 (16)	129 (17)	132 (18)	<0.001
Diastolic blood pressure, mean (SD), mmHg	83 (9)	83 (9)	84 (9)	85 (10)	<0.001
Resting heart rate, mean (SD), bpm	72 (7)	72 (7)	74 (8)	77 (9)	<0.001
Total cholesterol, mean (SD), mmol/L	5.0 (1.4)	5.0 (1.2)	5.0 (1.2)	5.0 (1.3)	0.075
High density lipoprotein, mean (SD), mmol/L	1.5 (0.5)	1.5 (0.5)	1.5 (0.5)	1.5 (0.5)	0.085
Fasting plasma glucose, mean (SD), mmol/L	5.6 (1.9)	5.6 (1.7)	5.6 (1.5)	5.8 (1.7)	<0.001
C-reactive protein >2 mg/L (%)	30	31	32	34	<0.001
Diabetes mellitus (%)	4	4	4	5	0.042
Hypertension (%)	42	43	44	48	<0.001
Antihypertensive medication drug class					
ACE inhibitor (%)	0.8	0.6	0.9	1.2	<0.001
ARB (%)	0.3	0.3	0.2	0.3	0.473
Calcium channel blocker (%)	1.0	1.0	1.0	1.3	0.080
Diuretic (%)	0.8	0.8	0.9	0.9	0.720
Other types (%)	9.5	9.4	10.4	12.2	<0.001

*Assessed using data at the 3^rd^ examination in the Kailuan Study (2010–2011). Continuous variables were presented as mean (SD). Categorical variables were expressed as percentages. bpm: beats per min. Differences between different heart rate quartiles at baseline were assessed using x ^2^ test for categorical variables and one-way ANOVA for continuous variables.

### Association between standard deviation of resting heart rate and clinical characteristics

Table 2 shows the association between RHR-SD and clinical characteristics. After adjusting for age, gender, BMI, current smoker category, mean SBP, mean RHR, FPG, total cholesterol, HDL, elevated CRP and ACE inhibitor, there were significant associations with older age (β = 0.09; *P* < 0.001), male gender (β = 0.48; *P* < 0.001), higher systolic blood pressure (β = 0.12; *P* < 0.001), higher mean heart rate (β = 1.64; *P* < 0.001), elevated C-reactive protein level (β = 0.09; *P* < 0.05) and ACE inhibitor medication (β = 0.54; *P* < 0.01) with RHR-SD.

**Table 2:** Standard deviation of resting heart rate associated with participant characteristics.

	Standard deviation of resting heart rate across examinations, bpm	
	Model 1*	Model 2^†^
Age, 10 years	0.07 (0.02)^‡^	0.09 (0.02)^‡^
Men	0.48 (0.05)^‡^	0.48 (0.05)^‡^
Obesity	0.12 (0.07)	…
Current smoker	−0.09 (0.04)^‖^	−0.21 (0.01)
High physical activity	−0.05 (0.06)	…
Systolic blood pressure, 20 mmHg	0.51 (0.03)^‡^	0.12 (0.02)^‡^
Diastolic blood pressure, 10 mmHg	0.37 (0.02)^‡^	−0.15 (0.04)^‡^
Resting heart rate, 10 bpm	1.64 (0.02)^‡^	1.64 (0.02)^‡^
Total cholesterol, 1 mmol/L	0.03 (0.02)^‖^	−0.02 (0.01)
High density lipoprotein, 1 mmol/L	0.06 (0.04)	…
Fasting plasma glucose, 1 mmol/L	0.09 (0.01)^‡^	−0.03 (0.01)
C-reactive protein >2 mg/L	0.26 (0.04)^‡^	0.09 (0.04)^‖^
Anti-hypertensive drugs		
ACE inhibitor	0.81 (0.20)^‡^	0.54 (0.20)^§^
ARB	0.06 (0.36)	−0.28 (0.34)
Calcium channel blocker	0.47 (0.18)^§^	0.29 (0.17)
Diuretics	0.30 (0.20)	…

Numbers in the table indicate β (standard error). All the characteristics were collected at the 3^rd^ examination in the Kailuan Study (2010–2011).

*Adjusted for age and gender.

^†^Adjusted for all variables associated with standard deviation of resting heart rate in Model 1.

^‡^ *P* < 0.001; ^§^ *P* < 0.01; ^‖^ *P* < 0.05.

### Overall mortality

**Figure 2:**
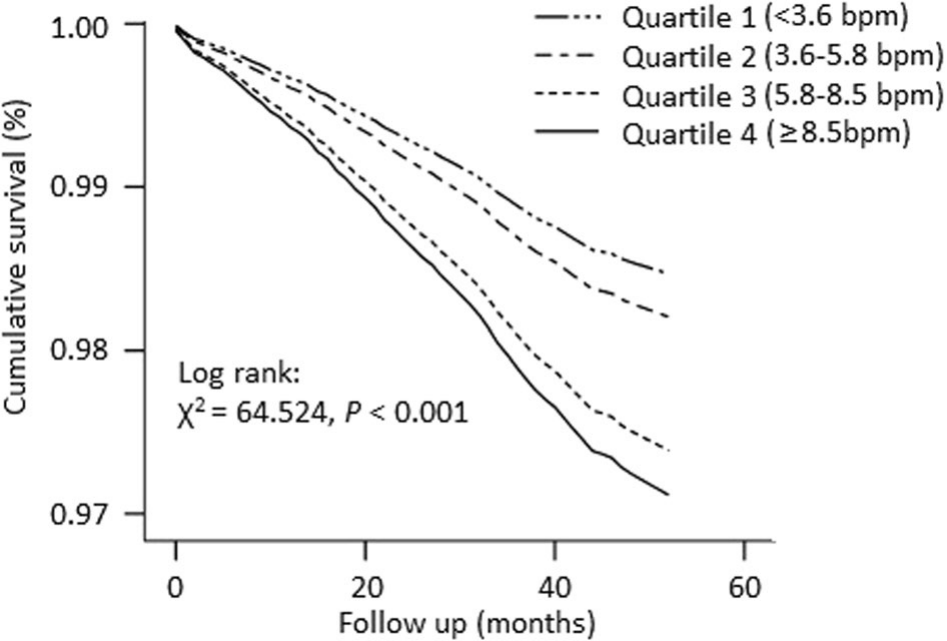
The Kaplan-Meier survival curves for all-cause mortality by quartiles of standard deviation of resting heart rate.

During a median follow-up period of 49.4 months, 973 participants died of all causes. Table 3 presents that all-cause mortality rates were 1.4%, 1.7%, 2.5%, and 2.7% for the quartile 1 to 4 of RHR-SD, 1.5%, 1.7%, 2.4%, and 2.7% for the quartile 1 to 4 of RHR-CV, and 1.3%, 1.8%, 2.4%, and 2.6% for the quartile 1 to 4 of RHR-ARV. Figure 2 displayed the Kaplan-Meier survival curves for all-cause mortality by quartiles of RHR-SD. Higher quartiles of RHR-SD were associated with a higher risk of total mortality during follow-up. After adjustment for all variables potentially associated with death, the higher quartiles of RHR-SD remained an independent predictor of all-cause mortality (Q3 versus Q1, HR = 1.51, 95% CI 1.24–1.84; Q4 versus Q1, HR = 1.43, 95% CI 1.18–1.74). Compared with the lowest RHR-CV group, the highest HR (95% CI) for the risk of all-cause death was 1.81 (95% CI, 1.50–2.18) in Model 1 and 1.57 (95% CI, 1.30–1.89) in Model 2, respectively (Table 3). In addition, there was still a significant relationship between RHR-CV and all-cause death after adjusting for all confounders in Model 3. Similar results were found when modeling RHR variation by ARV.

**Table 3:** Cumulative mortality and hazards ratios (95% confidence interval) for all-cause mortality associated with quartile of standard deviation (SD), coefficient of variation (CV), and average real variability (ARV) of resting heart rate (RHR).

	Quartile of RHR-SD, bpm				*P* for trend
	Quartile 1 (<3.6)	Quartile 2 (3.6–5.8)	Quartile 3 (5.8–8.5)	Quartile 4 (≥8.5)	
Total, n	11,676	12,230	10,912	12,055	—
Deaths, n (%)	168 (1.4)	207 (1.7)	269 (2.5)	329 (2.7)	<0.001
Model 1*	1.00 (Ref)	1.18 (0.96–1.45)	1.73 (1.42–2.09)	1.91 (1.58–2.30)	<0.001
Model 2^†^	1.00 (Ref)	1.17 (0.95–1.43)	1.64 (1.36–1.99)	1.72 (1.43–2.07)	<0.001
Model 3^‡^	1.00 (Ref)	1.16 (0.94–1.43)	1.51 (1.24–1.84)	1.43 (1.18–1.74)	<0.001
	**Quartile of RHR-CV, %**				
	**Quartile 1 (<5.0)**	**Quartile 2 (5.0–7.9)**	**Quartile 3 (7.9–11.7)**	**Quartile 4 (≥11.7)**	***P*** **for trend**
Total, n	11,471	11,795	11,742	11,693	—
Deaths, n (%)	172 (1.5)	206 (1.7)	284 (2.4)	311 (2.7)	<0.001
Model 1*	1.00 (Ref)	1.19 (0.97–1.45)	1.65 (1.37–2.00)	1.81 (1.50–2.18)	<0.001
Model 2^†^	1.00 (Ref)	1.16 (0.95–1.42)	1.55 (1.28–1.88)	1.57 (1.30–1.89)	<0.001
Model 3^‡^	1.00 (Ref)	1.16 (0.95–1.43)	1.49 (1.23–1.81)	1.41 (1.16–1.71)	<0.001
	**Quartile of RHR-ARV, bpm**				
	**Quartile 1 (<4.0)**	**Quartile 2 (4.0–7.0)**	**Quartile 3 (7.0–11.0)**	**Quartile 4 (≥11.0)**	***P*** **for trend**
Total, n	9,337	13,092	12,312	12,132	—
Deaths, n (%)	125 (1.3)	237 (1.8)	292 (2.4)	319 (2.6)	<0.001
Model 1*	1.00 (Ref)	1.36 (1.08–1.67)	1.77 (1.44–2.18)	1.96 (1.59–2.41)	<0.001
Model 2^†^	1.00 (Ref)	1.32 (1.06–1.63)	1.66 (1.35–2.05)	1.79 (1.45–2.20)	<0.001
Model 3^‡^	1.00 (Ref)	1.23 (0.99–1.54)	1.50 (1.21–1.86)	1.43 (1.16–1.78)	0.001

*Unadjusted model.

^†^Adjusted for age and gender.

^‡^Adjusted for age, gender, body mass index, current smoker, mean systolic blood pressure, mean resting heart rate, fasting plasma glucose, total cholesterol, high density lipoprotein, elevated C-reactive protein and ACE inhibitor.

**Figure 3:**
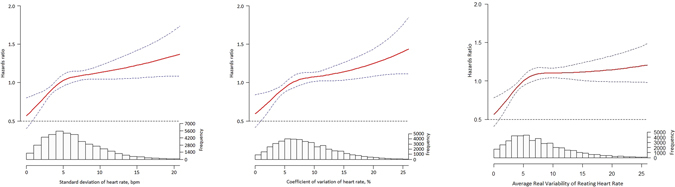
Association between standard deviation (left panel), coefficient of variation of resting heart rate (middle panel), and average real variability (right panel) with all-cause mortality over a median of 49.4 months follow-up.

Modeled as a continuous variable, the multivariable adjusted heart rate increased progressively across the full range of RHR-SD and kept statistically significant after 6 bpm (Fig. 3, left panel). The trend for the multiple-adjusted HR was consistent in RHR-CV (Fig. 3, middle panel) and RHR-ARV (Fig. 3, right panel).

## Discussion

The main finding of this contemporary population-based prospective cohort study is a positive and continuous association between RHR variation and the endpoint event (death). The elevated long-term RHR variation was an independent marker of subsequent death in the Kailuan Chinese community, and the risk of all-cause mortality was confirmed when repeating the analyses for multiple factors. While the positive relationship between elevated RHR and the risk of death has been well documented in the previous Kailuan study^10^, the present study added that RHR variation increases the risk of death. The findings of our study are consistent with the previous studies that reported a positive association between RHR variation and mortality^8, 11, 12^.

A prospective study conducted on patients without known cardiovascular disease reported that a greater increase in RHR over 10 years was associated with a greater risk of death^13^. Therefore, the increase in the change of RHR over the previously specified interval correlates with subsequent all-cause mortality (independent of other risk factors). RHR has also been reported to be associated with cardiovascular outcomes in patients with hypertension, heart failure and myocardial infarction^14–16^. However, the relationship between RHR variation over time and the clinical outcomes has been seldom investigated. Several investigations have identified that elevated RHR variation was associated with cardiovascular and total mortality by using a day-by-day measurement. For example, Kikuya *et al*. demonstrated that higher day-by-day heart rate variation was associated with cardiovascular and cardiac mortality^11^. Day-by-day RHR variation is the so-called “long term variation”, which differs from the variation over years. Recently, a study claimed that elevated RHR variation over 4 years was associated with an increased risk of myocardial infarction and death among older adults^8^; but it was a small sample size research that focused on the elderly population which could not be extrapolated to include the whole population. In the present investigation, the mortality risk was found to increase with greater RHR variation in the general population. Therefore, the identification of the prognostic value of long term RHR variation in the present study calls for the attention to the unusual fluctuation of heart rate during healthcare visits to prevent fatal outcomes.

The findings of this investigation regarding the long term RHR variation contrast with previously established findings of short term heart rate variability (HRV). Reduced short term HRV is an established prognostic marker for mortality and cardiovascular disease among the middle-aged^17^, the elderly^18^ population and the subjects with a variety of conditions including coronary heart disease^19^ and congestive heart failure^20^. However, short term HRV is based on time domain analysis, geometric methods, and frequency domain analysis^21^, which is distinct from long term RHR variation. Short term HRV is a mirror reflecting the activity of the sympathetic and vagal components of the autonomic nervous system on the sinus node of the heart^22^. Whereas, the influencing factors and clinical values of long term RHR variability are ambiguous.

The underlying mechanism of the relationship between higher RHR variation and the effect of long-term RHR variation on all-cause mortality is not well established. In addition to the report that revealed elevated heart rate was associated with atherosclerosis and endothelial dysfunction in both experimental animal models and in humans^23, 24^, recent investigations have demonstrated that inflammation is an important cause of cardiovascular diseases^25–27^. Also, the results from this study show that increased CRP level is closely linked to higher RHR variation (Table 2). This finding is in accordance with the study that reported a high level of high-sensitivity CRP associated with increased RHR^28^. Participants with higher RHR variation in the present study were linked to higher SBP, higher average RHR, and higher CRP level, which may be in response to activation of the sympathetic nervous system. Moreover, older age together with higher SBP was independently associated with greater variation of RHR in our study. Collectively, these findings suggest that both inflammation and hypertension, as well as activation of sympathetic nervous system contribute to greater RHR variation which eventually lead to increased all-cause mortality.

This study has several strengths including, the prospective design; large sample size; enrollment of men and women with a wide range of age (18–98 years old); confirmation of death events through reviewing medical records and complete follow-up for mortality; and the application of different methods of RHR variation models. However, some potential limitations of the present study should be taken into account. Echocardiography examinations to assess the cardiac function for congestive heart failure (CHF) were not performed. The Kailuan study was based on the workers from the Kailuan Company, most of whom were coal miner workers. Therefore, female enrollment was lower than males with an approximate male to female ratio of 3:1. In addition, the limited sample size of participants taking any antihypertensive medication may influence the results of the regression analysis. Finally, heart rate was measured at only 3-time points to measure the variability. It would be worthwhile to calculate variability with more visits spanning over an extended interval.

## Conclusion

In this large, general population-based study of all-cause mortality, RHR variation was independently associated with the increased risk for mortality. Although a causal association cannot be established, these findings support a significant relationship between RHR variation and all-cause mortality, independently from mean RHR and other clinical covariates. Further investigations are needed to confirm these findings, identify the mechanisms involved in this association, and evaluate approaches to reduce long-term variability in RHR.
